# High quality protein microarray using *in situ *protein purification

**DOI:** 10.1186/1472-6750-9-72

**Published:** 2009-08-23

**Authors:** Keehwan Kwon, Carissa Grose, Rembert Pieper, Gagan A Pandya, Robert D Fleischmann, Scott N Peterson

**Affiliations:** 1 Pathogen Functional Genomics Resource Center, J. Craig Venter Institute, 9704 Medical Center Drive, Rockville, Maryland 20850, USA

## Abstract

**Background:**

In the postgenomic era, high throughput protein expression and protein microarray technologies have progressed markedly permitting screening of therapeutic reagents and discovery of novel protein functions. Hexa-histidine is one of the most commonly used fusion tags for protein expression due to its small size and convenient purification via immobilized metal ion affinity chromatography (IMAC). This purification process has been adapted to the protein microarray format, but the quality of *in situ *His-tagged protein purification on slides has not been systematically evaluated. We established methods to determine the level of purification of such proteins on metal chelate-modified slide surfaces. Optimized *in situ *purification of His-tagged recombinant proteins has the potential to become the new gold standard for cost-effective generation of high-quality and high-density protein microarrays.

**Results:**

Two slide surfaces were examined, chelated Cu^2+ ^slides suspended on a polyethylene glycol (PEG) coating and chelated Ni^2+ ^slides immobilized on a support without PEG coating. Using PEG-coated chelated Cu^2+ ^slides, consistently higher purities of recombinant proteins were measured. An optimized wash buffer (PBST) composed of 10 mM phosphate buffer, 2.7 mM KCl, 140 mM NaCl and 0.05% Tween 20, pH 7.4, further improved protein purity levels. Using *Escherichia coli *cell lysates expressing 90 recombinant *Streptococcus pneumoniae *proteins, 73 proteins were successfully immobilized, and 66 proteins were *in situ *purified with greater than 90% purity. We identified several antigens among the *in situ*-purified proteins via assays with anti-*S. pneumoniae *rabbit antibodies and a human patient antiserum, as a demonstration project of large scale microarray-based immunoproteomics profiling. The methodology is compatible with higher throughput formats of *in vivo *protein expression, eliminates the need for resin-based purification and circumvents protein solubility and denaturation problems caused by buffer exchange steps and freeze-thaw cycles, which are associated with resin-based purification, intermittent protein storage and deposition on microarrays.

**Conclusion:**

An optimized platform for *in situ *protein purification on microarray slides using His-tagged recombinant proteins is a desirable tool for the screening of novel protein functions and protein-protein interactions. In the context of immunoproteomics, such protein microarrays are complimentary to approaches using non-recombinant methods to discover and characterize bacterial antigens.

## Background

Sharp increases in the efficiency of DNA sequencing technology, coupled with dramatic decreases in cost have led to very large quantities of DNA sequences to be deposited into public databases. The number of complete genomes continues to grow. More recently microbial DNA sequences were derived from the environment and those living in the context of the human host. Taken together the numbers of genes of unknown function as well as those without experimental confirmation grow at unprecedented rates. These advances have generated a strong need for the development of very high throughput methodologies with the capacity to characterize protein functions in a highly parallel manner.

In the post-genomic era, highly parallel functional analysis of proteins including hypothetical and conserved hypothetical proteins has emerged as a major research interest. Protein microarrays represent one of the most powerful tools in this context [1-4]. Technical approaches for protein microarrays have benefitted from its predecessor, DNA microarray technology. Protein microarrays are designed for high throughput analysis of proteins using very small quantities of purified proteins [5]. Various protein microarray platforms have been developed, not only for screening antibodies and antigens but also for discovery of novel enzymes and enzymatic activities, protein-ligand, protein-nucleic acid and protein-protein interactions [6-11]. Immunoproteomic analyses, that assess antibody-antigen interactions, are among the most established microarray applications [12-15]. Theses assays have the potential to impact the field of infectious disease diagnostics and greatly facilitate the design of subunit vaccines [12,14]. The development of microarray applications for the functional characterization of proteins of unknown or unproven function continues to be a major challenge [15-18].

The generation and immobilization of pure protein reagents are critical to the quality of protein microarrays. Many high throughput protein production platforms have been reported [19-25]. Proteins are typically overexpressed in a heterologous host system (*E. coli*) and purified via affinity chromatography to fusion tags associated with the recombinant proteins. A recently introduced approach is based on highly parallel *in vitro *transcription and translation using cellular extracts [26-28]. While powerful, the difficulty with column based recombinant protein purification is relate to reduced throughput potential and low protein stability and solubility of proteins during pre-immobilization processing steps. The *in vitro *transcription and translation approach is relatively expensive and requires slide surfaces suitable for the immobilization of DNA and proteins. Recently, the technology has improved in efficiency and made significant studies with regard to long term instability issues of proteins prior to immobilization [9,29-31]. An *in situ *expression and purification method for GST-tagged proteins was reported by Ramanchandran *et al*. [9,32].

On-chip purification of recombinant His-tagged proteins on metal chelate affinity surface chips has been described [31,33-35]. The quality of the on-chip purification method has not been systematically tested. Moreover, a major concern is that microarray chip surfaces allow non-specific binding of endogenous cellular proteins. To address these issues, double His-tagged fusion proteins permitting more stringent purification conditions were introduced [33,36].

Recently, we developed a high throughput His-tagged protein production pipeline in *E. coli*, based on the Gateway cloning and expression system [24]. Here, we report a platform that combines recombinant protein expression in *E. coli *with *in situ *protein purification using a chip surface with a low propensity to bind proteins non-specifically. We compare the quality of the resulting protein microarray with that of a conventional metal chelate surface and assess purity and recognition of the proteins by serum antibodies.

## Results and Discussion

### Technical approach for *in situ* protein purification on metal chelate surface microarray slides

**Figure 1:**
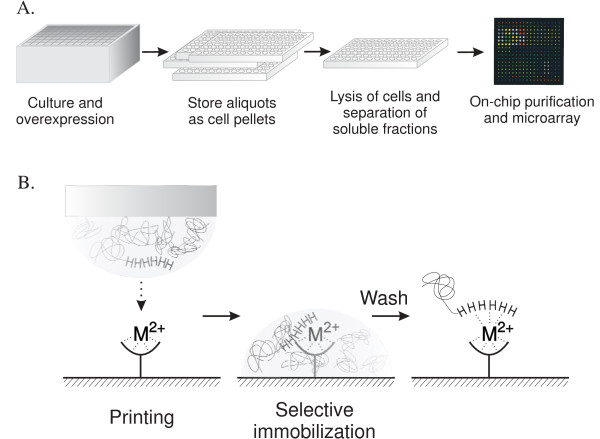
**Schematic of *in situ *purification of His-tagged proteins on protein microarray slides**. A) Flow diagram from recombinant protein expression to *in situ *purification and protein microarray. Cells were cultured in 1 mL 2xYT media in a 96-well block and the target proteins were overexpressed by adding IPTG at O.D._600 nm _= 0.7–0.9. After induction, the cells were divided into several 96-well plates, and the cell pellets were stored at -20°C. The cell pellets were resuspended, and lysed. Soluble fractions of lysates were subjected to *in situ *purification. Metal ions, either Ni^2+ ^or Cu^2+^, were attached to the surface of a chip with a chelate structure. The chip surface was blocked with PBS buffer containing 5 mM imidazole and 3% dry milk, and the *E. coli *endogenous proteins were washed off with PBST, pH 7.4. The chips can be either stored or applied for protein microarray application directly. B) Scheme of selective immobilization of recombinant proteins. Approximately 50 nL of each soluble fraction of cell lysates was spotted on the metal ion (Ni^2+ ^or Cu^2+^) chelated chip. His-tagged recombinant proteins were immobilized by binding tightly to the metal chelate. The endogenous *E. coli *proteins were washed with PBS containing 0.05% Tween 20.

*In situ *protein purification on metal chelate chips synchronizes protein purification and immobilization significantly reduces time, effort and cost of post-protein expression processes, and generates high quality protein microarray chips reproducibly. The approach we present here can be combined with any expression system that yields fusion proteins with an N- or C-terminal hexa-histidine tag (His-tag). The same source set of expressed proteins allows routine reproduction of identical protein microarray chips. Both the microarray replicates and aliquots of cells expressing the recombinant proteins can be stored long-term without major concerns about the quality of the assays performed subsequently. This setup circumvents the problems caused by the long-term storage of purified proteins, which include protein degradation, precipitation and aggregation. Buffer exchange of purified and stored proteins at -80°C is not required. A flow diagram for *in situ *protein purification from *E. coli *cell cultures is shown in Figure 1. Recombinant proteins were expressed in *E. coli *in 96-well block. Cell cultures were divided into aliquots in 96-well plates and stored as cell pellets at -80°C. The cell aliquots were subjected to lysis followed by *in situ *purification and immobilization of proteins via specific interactions of His-tagged proteins with a slide surface chelated with either Ni^2+ ^or Cu^2+ ^(Figure 1B). High affinity interactions allow relatively stringent wash steps in which the majority of weakly bound proteins are removed. Protein microarrays are stored at 4°C or used directly.

### Optimization of *in situ* protein purification

Purity and quantity of *in situ *purified recombinant proteins are highly dependent on a number of factors, including the slide surface chemistry, solutions used for printing slides and washing steps, the relative concentration of recombinant proteins to host endogenous proteins, recombinant protein solubility and the ambient humidity during immobilization. There are two different types of protein impurities, one related to non-specific binding and the other caused by competitive binding. Non-specific interactions of endogenous *E. coli *proteins with a metal chelate surface depend primarily on the chemistry of the support surface. These impurities can be reduced or eliminated using effective wash steps. In the context of His-tagged proteins, competitive binding is often linked to histidine-rich *E. coli *proteins such as the FKBP-type peptidyl-prolyl-cis-trans-isomerase SlyD. Such impurities cannot be eliminated easily and are independent on the immobilization support. The higher an expression level of a recombinant protein, the lower the competitive binding effect of host endogenous proteins. In this study, we examined wash buffers to minimize protein impurities resulting from non-specific binding.

Two His-tagged recombinant proteins, the *E. coli *Lac repressor (LacI) and the *S. pneumoniae *GTP cyclohydrolase I (FolE) were used to determine the optimum buffers for printing and washing on two commercially available slide supports. The first solid support binds a His-tag using Ni^2+ ^chelated by nitrilotriacetic acid (Ni^2+^/NTA) and the second using Cu^2+ ^by iminodiacetic acid groups on a polyethylene glycol coated surface (Cu^2+^/IDA/PEG). We used a quantitative approach to discern specific binding of His-tagged proteins to the reactive surface from non-specific binding of endogenous *E. coli *proteins. The relative quantity of immobilized His-tagged recombinant proteins was measured with an Alexa Fluor 555 conjugated anti-His-tag antibody, and the impurities were measured using a rabbit anti-*E. coli *antibody derived with a mixture of the *E. coli *serotypes O and K. The antibody-antigen reactions were visualized using an ATTO 550 conjugate to anti-rabbit or anti-human IgG as a secondary antibody. Relative purities of immobilized His-tagged proteins on the slides were estimated using the following equation.

(1)

**Figure 2:**
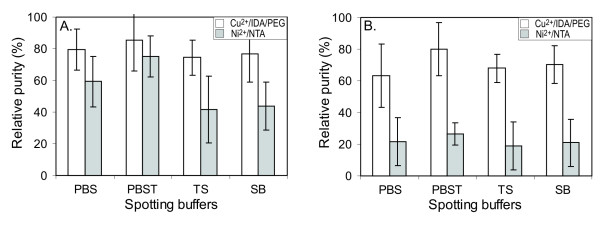
**Optimization of *in situ *protein purification**. (A) His-tagged FolE and (B) His-tagged Lac repressor. Relative purities of *in situ *purified proteins were determined using the ratio of fluorescence signals of recombinant proteins versus the fluorescence signals of total protein, as shown in Equation 1 (see text). White and gray bars represent Cu^2+^/IDA/PEG slides and non-PEG coated Ni^2+^/NTA slides, respectively. A series of spotting buffers were examined: PBST [10 mM phosphate buffer, 2.7 mM KCl, 140 mM NaCl, 0.05% Tween 20, pH 7.4], PBS [10 mM phosphate buffer, 154 mM NaCl, pH 7.4], TS [50 mM Tris, 300 mM NaCl, 10 mM imidazole, pH 7.8] and SB [100 mM sodium borate, pH 7.8].

The calculated relative purities were used as criteria to define optimal conditions for *in situ *purification. Instead of using two-channel detection method, one-channel detection method for His-tagged proteins and *E. coli *endogenous protein contaminants on a 2-pad slide system was used to eliminate the mutual inferences of two antibody interactions to the immobilized proteins and to keep the same background in scanning. Considering the fluorescence brightness of Alexa Fluor 555 and ATTO 550, we estimate that the true level of purity of the immobilized recombinant proteins is greater than the relative purity measured according to Equation 1. The fluorescence brightness, which is a product of an extinction coefficient and a quantum yield of the fluorophore, of Alexa Fluor 555 and ATTO 550 are 15,000 and 96,000, respectively [37]. Using GenePix 4000B image scanner and identical experimental conditions, the brightness of ATTO 550 is at least as high as to that of Alexa Fluor 555. Combinations of four spotting buffers, PBS, PBST, TS and SB and three washing buffers, PBS, PBST and SB were examined for *in situ *purification of FolE and LacI. Based upon the estimation of the relative purity levels of FolE and LacI, PBST delivered the best results as a spotting and wash buffer for both Cu^2+^/IDA/PEG and Ni^2+^/NTA slides. In Figure 2, relative purities of FolE and LacI, printed with four spotting buffers on both slide surfaces and washed with PBST, were compared. The relative purities of FolE with two spotting buffers, PBS and PBST were similar but the relative purity of Lac I with PBST spotting buffer was nearly 20% higher than that with PBS. Apparently, the nonionic detergent Tween-20 mediates partial denaturation of recombinant proteins and enhances the exposure of His-tags to the Cu^2+ ^or Ni^2+ ^on the slides. This effect may also increase competitive binding of recombinant proteins to histidine-rich contaminant *E. coli *proteins.

The binding and purification characteristics of the two slide supports, Cu^2+^/IDA/PEG and Ni^2+^/NTA, were evaluated using relative purity levels of LacI and FolE (Figure 2). Relative purities of LacI and FolE with PBST were approximately 80% and 85%, respectively, on the Cu^2+^/IDA/PEG slide, and approximately 35% and 75%, respectively, on the Ni^2+^/NTA slides. His-tagged FolE purified with higher relative purity than the recombinant LacI on both slides. Interestingly, relative purities of proteins using the *in situ *purification method were correlated to the quantities of the corresponding solubly expressed proteins in the *E. coli *cell lysate. His-tagged FolE expressed solubly at approximately 4.5-fold greater abundance than His-tagged LacI. The difference in relative purity levels of the two recombinant proteins was much greater on the Ni^2+^/NTA slide (45%) than on the Cu^2+^/IDA/PEG slide (10%). Apparently, non-specific protein adsorption on the slide surface reduced the purity level. Lower non-specific protein adsorption resulted in less variation in immobilized protein purity. This finding indicates that the consistency of *in situ *purification of proteins is dependent not only on the surface chemistry of the slide but also on the quantity of the solubly expressed recombinant proteins. It is clear that purity and consistency of *in situ *purification using Cu^2+^/IDA/PEG slides are superior to that using Ni^2+^/NTA chelated slides.

The performance of both slide supports was also evaluated with purified LacI as well as FolE and LacI in the *E. coli *lysate supernatants. The immobilization of purified LacI on the slides is not interfered by *E. coli *endogenous proteins. We determined that the same relative purity levels were achieved with pre-purified LacI compared to unpurified LacI, independent of the slide support. However, the fluorescence intensity for the immobilized LacI on the Cu^2+^/IDA/PEG was about 1.9-fold greater than that on the Ni^2+ ^slide. When *E. coli *lysate containing overexpressed recombinant proteins, either FolE or LacI, was applied on the slides, non-specific immobilization of *E. coli *endogenous proteins on Ni^2+^/NTA was at least 3-fold more than on Cu^2+^/IDA/PEG. Lower capacity of the Ni^2+^/NTA slides could be an effect of reduced affinity, density of reactive groups on slide surface or less accessibility of His-tag by the antibody. The much higher nonspecific interactions of Ni^2+^/NTA resulted from a difference in the support chemistry and lack of PEG coating. Since the lower fluorescence signal for purified LacI on the Ni^2+^/NTA compared to the Cu^2+^/IDA/PEG was observed, the much lower *E. coli *endogenous signal on Cu^2+^/IDA/PEG is more likely due to the PEG coating which has been reported and used to prevent nonspecific interactions [38-40].

**Figure 3:**
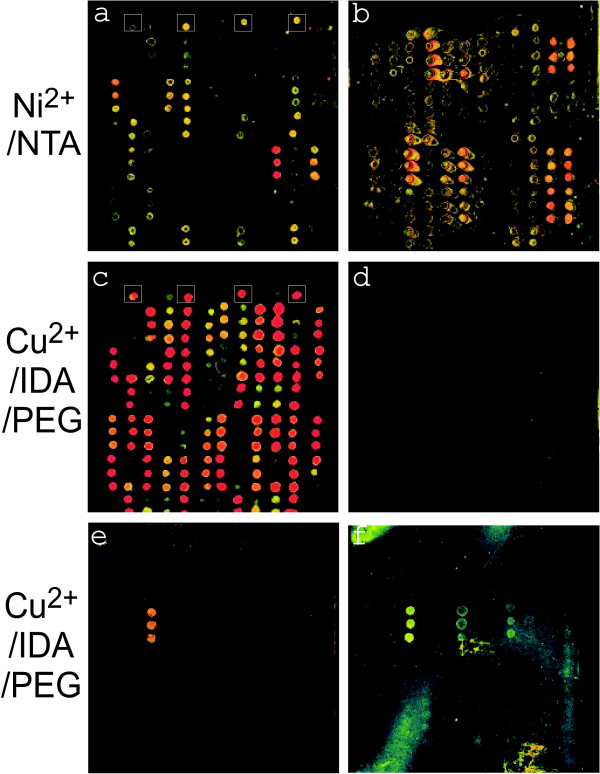
**Application of *in situ *purified *S. pneumoniae *proteins to immunoassays**. The overexpressed proteins were manually printed at approximately 70% humidity using the MicroCaster manual printing device (Whatman, Florham Park, NJ). Immobilized proteins were *in situ *purified and quantities were assessed using an Alexa Fluor 555 conjugated anti-His-tag antibody (a, c). Small white squares in a) and c) denote the position of the immobilized *E. coli *Lac repressor (control). The relative purity of His-tagged *S. pneumoniae *proteins was assessed by assays with an anti-*E. coli *antibody and an ATTO 550 labeled 2^nd ^antibody (b, d) which resulted in the detection of endogenous *E. coli *proteins where His-tagged proteins were impure. The protein microarray on the Cu^2+^/IDA/PEG chip (c, d) was subjected to a detection assay with a rabbit anti-*S. pneumoniae *antibody followed by a 2^nd ^antibody (labeled with ATTO 550) (e) and with a human patient serum followed by a 2^nd ^goat anti IgG antibody (labeled with Alexa Fluor 555) (f).

Both slide types were further examined with 90 recombinant *S. pneumoniae *proteins present in *E. coli *cell lysates in varying concentrations. As shown in Figure 3b and 3d, *E. coli *endogenous proteins were frequently visualized as contaminants on the Ni^2+^/NTA slide surface, but rarely on the Cu^2+^/IDA/PEG slide surface. Differences in Alexa Fluor 555 conjugated anti-His-tag antibody signal intensities (Figure 3a and 3c) suggest that the slide surface strongly influenced the efficiency of immobilization of His-tagged proteins. The fluorescence intensities representing protein concentrations (F_His-tag_) varied considerably among the 90 *S. pneumonia *proteins (Additional file 1). In summary, this data supported the conclusions made based on the data with LacI and FolE. The Cu^2+^/IDA/PEG is technically clearly superior to the Ni^2+^/NTA.

### Purification and solubility characteristics of 90 recombinant *S. pneumoniae* proteins

Randomly selected 90 *S. pneumoniae *proteins were categorized as membrane and non-membrane proteins using HMM, INTERPRO domain searches, GO annotations and COG analysis, in addition to transmembrane domain motif searches with TMHMM [41], SOSUI [42] and HMMTOP [43]. Thirty-three proteins were predicted to be membrane proteins. An overview of the *in situ *purification of these proteins is provided in Table 1. More than 80% of the non-membrane proteins were purified, and 96% of them were calculated to have a purity level higher than 90%. Interestingly, nearly 73% of the membrane proteins were also purified and 79% of them surpassed the 90% purity level. Membrane protein purification is known to be challenging. In our previous study using this protein expression strategy, but a different quantitative detection system, the S-tag [24], more than 50% of the membrane proteins were solubly expressed, 60% of which were purified using a Ni^2+^/NTA agarose resin. Although our sample numbers were limited for *in situ *purification (33 proteins), a trend towards more effective membrane protein purification on a slide surface is apparent. A study on *in vitro *expressed C-terminal GST-tagged proteins, which were obtained by the nucleic acid programmable protein array (NAPPA) method, reported a 93% detection level for membrane proteins [9], and supported our conclusions. The fact that many membrane proteins have domains exposed at the cell surface, makes the ability to effectively purify them important in the context of their antigenicity and role as vaccine candidates for human pathogens.

**Table 1:** Summary of success frequencies and purities of the *in situ *purification of 90 recombinant *S. pneumoniae *proteins.

Protein class	Number of proteins	Purification (%)	>90% relative purity (%)	<90% relative purity (%)
Membrane	33	24 (72.7%)	19 (57.6%)	5 (15.2%)

Non-membrane	57	49 (86.0%)	47 (82.5%)	2 (3.5%)

Total	90	73 (81.1%)	66 (73.3%)	7 (7.8%)

Immobilized His-tagged proteins and *E. coli *endogenous proteins were detected using an Alexa Fluor 555 fluorophore conjugated anti-His-tag antibody, and rabbit anti-*E. coli *antibody followed by incubation with ATTO 550 conjugated goat anti-rabbit antibody. The relative purity was calculated using the Equation 1 (text).

### Identification of immunogenic *S. pneumoniae* proteins

The protein microarray platform was used for a pilot project, immunoproteomic analysis of the Gram-negative bacterial pathogen, *S. pneumoniae*. Anti-*S. pneumoniae *antibodies raised in rabbits using a mixture of nine *S. pneumoniae *serotypes as immunogens and an antiserum of a patient infected with *S. pneumoniae *serotype IV were used. The antibodies and antisera were pre-treated with *E. coli *cell extracts permitting removal of *E. coli *protein-reactive antibodies which are known to circulate in most animal and human sera. Binding affinities of anti-*S. pneumoniae *antibodies to recombinant proteins were determined using the following equation.

(2)

R_F _in Eq. 2 represents fluorescence intensity ratio of a labeled secondary antibody that measures the interaction of anti-*S. pneumoniae *antibodies with immobilized proteins, to the Alexa Fluor 555 conjugated anti-His-tag antibody. The anti-His-tag antibody served as a normalizing factor to compare antigen recognition activities. This is useful because different proteins are immobilized in different quantities. Three types of R_F _values for a rabbit anti-*S. pneumoniae *antibody (R_Ab_), a human patient antiserum (R_S1_) and a healthy human serum (R_S0_), are provided in the Additional file 1. R_F _values greater than 1 suggested an interaction between a protein and anti-*S. pneumoniae *antibodies. A strongly reactive protein in the assay with rabbit antibodies was the manganese ABC transporter adhesion lipoprotein (PsaA) (Figure 3e). This lipoprotein is involved in pathogenicity via its role as a host cell adhesion protein. It is a highly conserved immunogenic protein among many of the 90 *S. pneumoniae *serotypes [44,45]. Data for the human patient antiserum (S_1_) were compared to those for a non-infected human serum (S_0_). Immunogenicity was defined by R_F _(>1) mentioned above and R (>2), a ratio of S_1 _to S_0_. In addition to PsaA, the iron-compound ABC transporter PiuA (SP1872) was also identified as a strong antigen. PiuA is suggested to bind extracellular iron and deliver it to the permease of the ABC transporter. The permease facilitates import of the cation into the cytoplasm. In *S. pneumoniae *(TIGR4), 10 proteins are annotated as iron ABC transporters. Although mechanisms of iron uptake by *S. pneumoniae *are not well characterized, iron transporters are known to be strong antigens and required for full virulence [46-48]. PsaA and PiuA are potential vaccine candidates and potential antigenic markers for the diagnosis of *S. pneumoniae *infections. Both proteins are anchored to the cytoplasmic membrane and exposed at the surface of *S. pneumoniae*. This study sets the stage for expression and immunogenic analyses of a larger number of ABC transporters and other cell surface-localized proteins, testing a large number of human patient sera. Another interesting application is the design of a microarray chip displaying a range of antigens recognized at various time points during an infection with *S. pneumoniae *and convalescence of the patient.

## Conclusion

To our knowledge, this is the first report describing a semi-quantitative strategy for the measurement of relative protein purities immobilized *in situ *on protein microarrays. A combination of antibodies, one measuring the target protein (a His-tagged recombinant protein), the other measuring the contamination level with endogenous *E. coli *proteins, was used. The strategy we employed has potential to become a new gold standard for high quality protein microarrays. We demonstrate that Cu^2+^/IDA/PEG successfully reduced non-specific adsorption of proteins on the substrate. Finally, we demonstrate that this protein microarray is useful for the discovery of immunogenic proteins of a bacterium that causes serious infections in humans.

## Methods

### Cloning and transformation

From the genome-wide cloning set of *Streptococcus pneumoniae*, TIGR4, previously described [24], 90 ORFs representing a variety of expression levels were selected for this study. Clones of the ORFs into pET-DEST-TIGR02 (T02) expression vector were transformed into BLR(DE3) cells (EMD Biosciences, San Diego, CA) using heat shock method. Transformants were plated on divided Q-trays containing 2xYT agar with 100 μg/mL ampicillin, 15 μg/mL tetracycline, 0.8% glucose and incubated at 37°C overnight. A single colony for each clone was picked into a deep well block containing 1 mL 2xYT, 100 μg/mL ampicillin, 15 μg/mL tetracycline, 0.8% glucose in each well. The deep well block was grown at 37°C in multitron shaker at 800 RPM until reaching OD_600 _0.7–0.9. The cultures were aliquoted to new microtiter plates and glycerol was added to 10% final concentration. The prepared frozen cultures were stored at -80°C.

### Protein overexpression

Cultures for overexpression were set in a 2 mL deep well block with 1 mL 2xYT broth containing 100 μg/mL ampicillin, 15 μg/mL tetracycline and 0.8% glucose. After inoculating with 20 μL frozen culture, the cultures were incubated at 37°C in a multitron shaker at 800 RPM until OD_600 _reached 0.7–0.9. Overexpression was induced by adding 10 μL freshly prepared 100 mM IPTG and plates were incubated overnight at 25°C, 800 RPM. After 18 hours incubation, 50 μL of culture were aliquoted to several microtiter plates. Cells were pelleted at 3,000 g for 5 min. The waste media was poured off and the pellets were stored at -80°C until ready for lysis.

### Lysis and S-tag assay

The frozen cell pellets were thawed on ice and resuspended in the 50 μL master mix of lysis buffer [50 mM Tris, 300 mM NaCl, 10 mM imidazole, 1 mM DTT pH 7.8 at 4°C] and Popculture and lysonase (EMD-Novagen). After complete resuspension of the cell pellets mixed by pipette, the plate was shaken at room temperature for 15 minutes to complete lysis. The supernatant of lysate was separated at 2,000 g for 20 minutes and transfer to a new 96-well plate. The quantities of recombinant proteins in the supernant were evaluated semi-quantitatively using S-tag assay as described previously [24].

### Printing and washing

Two slides, Ni^2+ ^chelated (Xenopore Corp., Hawthorne, NJ) and Cu^2+ ^chelated (MicroSurfaces, Inc., Minneapolis, MN) surface slides were examined. The slides were incubated in a room temperature humidity chamber for thirty minutes prior to printing. The lysate supernatants were diluted 1:20 in printing buffers. Slides were manually printed at ~70% humidity using the MicroCaster manual printing device (Whatman, Florham Park, NJ). Each supernatant was printed in triplicate on a single slide along with supernatant obtained from BLR(DE3) without expression clones for negative control. After printing, spots were allowed to dry at ~70% humidity. Combinations of a printing buffers PBST [10 mM phosphate buffer, 2.7 mM KCl, 140 mM NaCl, 0.05% Tween 20, pH 7.4], PBS [10 mM phosphate buffer, 154 mM NaCl, pH 7.4], TS [50 mM Tris, 300 mM NaCl, 10 mM imidazole, pH 7.8] and SB [300 mM sodium borate,, pH 7.8] and washing buffers: PBST, PBS and SB, were examined with *E. coli *superanatant containing recombinantly expressed FolE and Lac repressor. After evaluating, PBST was used for printing and washing steps *in situ *purification of 90 His-tagged proteins in *S. pneumoniae*.

### Protein microarray with antibody and antiserum

The printed slides were placed in Fast Frame incubation chambers (Whatman, Florham Park, New Jersey) and blocked with PBS, 5 mM imidazole pH 7.4, 3% dry milk, for 1 hour. The blocking solution was drawn off and slides were washed three times with 2 mL milliQ water each time. The immobilized recombinant proteins were visualized and quantified by reaction with 200 ng/mL Alexa Fluor 555 labeled Penta-His antibody in PBS (Qiagen, Valencia, CA). After 1 hour incubation, the slide was washed twice with fresh washing buffer (PBST) and rinse with milliQ water. The protein chip was subject to rabbit anti-*E. coli *antibody (Meridian Life Science, Inc., Saco, ME) and goat anti-rabbit antibody labeled with ATTO 550 to detect of contamination of *E. coli *endogenous proteins after purification. The chip was also applied to anti-*S. pneumoniae *rabbit antibody (Meridian Life Science, Inc., Saco, ME) and human patient antiserum. The rabbit antibody was diluted to a final concentration of 1.6 μg/mL in PBS and applied on the protein chip. After incubation for 1 hour, the chip was washed three times with washing buffer. ATTO 550 labeled anti-rabbit goat antibody was diluted 1:1000 in 1 mL PBS was applied on the slide in and incubated at room temperature in the dark for 1 hour. The slide was washed as described for Penta-His antibody reaction. Human patient serum was prepared 1:250 dilution in PBS. The reaction procedure was identical to anti-*S. pneumoiae *antibody reaction except using 1 μL Alexa Fluor 555 labeled anti-human IgG goat antibody. The slides were washed twice with fresh washing buffer and rinse with milliQ water. The slide was scanned using a GenePix4000B (Molecular Devices, Sunnyvale, CA). A GenePix laser of 532 nm and an emission filter of 557–592 nm were used to obtain fluorescence image.

All antibodies except anti-*E. coli *antibody and antiserum were treated with *E. coli *cell lysate to eliminate anti-*E. coli *antibody. A 1 mL BLR(DE3) cell pellet was lysed with 100 uL sodium phosphate lysis solution at room temperature for 15 minutes. Five microliters of whole BLR(DE3) lysate was added to the diluted primary and secondary antibody and incubated at room temperature for 30 minutes. The cell debris was pelleted at 2,000 × g for 20 minutes. The supernatants were transferred to a new microfuge tube for immunoassays.

### Analysis

All images were analyzed using Spotfinder. .

## Competing interests

The authors declare that they have no competing interests.

## Authors' contributions

KK, designed experiments, developed in situ purification strategy, interpreted the data, wrote the manuscript; CG, performed in situ purification experiments, optimized protein immobilization conditions, analyzed data; RP, participated in the study design, manuscript development and its review; GP, participated in the review of the study; RDF, participated in the design of the study; SNP, participated in the design of the study and the review of the manuscript.

## Supplementary Material

Additional file 1 **Immunoassay data were obtained from an *in situ *protein microarray with 90 *S. pneumoniae *proteins**. The S-tag assay scores are correlated with the quantities of recombinant proteins in the soluble fraction after cell lysis, therefore the scores represent the solubility of the recombinant proteins (Kwon *et al*., 2007). The values of H(F_His-tag_) represent fluorescence intensities from a labeled anti-His-tag antibody. Values of E(F_*E. coli*_) and S(F_*Sp*_) were obtained from fluorophore-labeled secondary antibodies, following binding of anti-*E. coli *and anti-*S. pneumoniae *antibodies, respectively. Values of S_1_(F_27_) and S_0_(F_48_) result from assays with a human patient antiserum and a healthy human antiserum, respectively. Click here for file
