# Supplementary material for: High quality protein microarray using in situ protein purification
Source: BMC Biotechnol. 2009 Aug 23;9:72. doi: 10.1186/1472-6750-9-72 (PMC2746808; doi:10.1186/1472-6750-9-72)
Supplement: Additional file 1 — Immunoassay data were obtained from an in situ protein microarray with 90 S. pneumoniae proteins. The S-tag assay scores are correlated with the quantities of recombinant proteins in the soluble fraction after cell lysis, therefore the scores represent the solubility of the recombinant proteins (Kwon et al., 2007). The values of H(FHis-tag) represent fluorescence intensities from a labeled anti-His-tag antibody. Values of E(FE. coli) and S(FSp) were obtained from fluorophore-labeled secondary antibodies, following binding of anti-E. coli and anti-S. pneumoniae antibodies, respectively. Values of S1(F27) and S0(F48) result from assays with a human patient antiserum and a healthy human antiserum, respectively. [file 1472-6750-9-72-S1.pdf]

Additional file 1.. Immunoassay data were obtained from an *in situ* protein microarray with 90 *S. pneumoniae* proteins.

Locus ID	Name of Protein	S-tag	H ($F_{His\text{-}tag}$)	E ($F_{E.coli}$)	S ($F_{S.p}$)	S_1 (F_{27})	S_0 (F_{48})	P (H/(E+H))	R_{Ab} (S/H)	R_{S1} (S_1/H)	R_{S0} (S_0/H)	R (S_1/ S_0)
SP0035	aromatic amino acid aminotransferase (araT)	4	55844282	0	0	0	0	1.00	0	0.00	0.00	-
SP0064	PTS system; IIA component	2	31633997	0	0	0	0	1.00	0	0.00	0.00	-
SP0079	potassium uptake protein, Trk family	2	20265235	1020550	0	0	0	0.95	0	0.00	0.00	-
SP0154	hypothetical protein	2	0	0	0	0	0	-	0	-	-	-
SP0168	macrolide efflux protein; putative	0	0	0	0	0	0	-	0	-	-	-
SP0236	DNA-directed RNA polymerase; alpha subunit (rpoA)	4	50494240	0	0	1225387	579909	1.00	0	0.02	0.01	2.1
SP0238	ACT domain protein	0	149001994	1998636	0	0	145467	0.99	0	0.00	0.00	0.0
SP0278	aminopeptidase PepS (pepS)	4	24646666	1287559	0	0	0	0.95	0	0.00	0.00	-
SP0280	ribosomal small subunit pseudouridine synthase A (rsuA-1)	2	0	0	0	0	0	-	0	-	-	-
SP0281	aminopeptidase C (pepC)	1	176626667	4152694	0	0	0	0.98	0	0.00	0.00	-
SP0291	GTP cyclohydrolase I (folE)	4	220350000	0	0	0	0	1.00	0	0.00	0.00	-
SP0308	PTS system; IIA component	3	369936667	1453692	0	0	0	1.00	0	0.00	0.00	-
SP0321	PTS system; IIA component	4	0	0	0	0	0	-	0	-	-	-
SP0360	UDP-N-acetylglucosamine 2-epimerase (cps4L)	3	141364860	0	0	0	0	1.00	0	0.00	0.00	-
SP0373	conserved hypothetical protein	4	155025000	3845625	0	1570478	1321411	0.98	0	0.01	0.01	1.2
SP0385	conserved hypothetical protein	1	251336	1451332	0	0	822425	0.15	0	0.00	3.27	0.0
SP0402	signal peptidase I (spi)	3	0	0	0	0	0	-	0	-	-	-
SP0422	3-oxoacyl-(acyl-carrier-protein) synthase II (fabF)	3	104004316	385479	0	0	0	1.00	0	0.00	0.00	-
SP0435	translation elongation factor P (efp)	4	44350039	0	0	0	0	1.00	0	0.00	0.00	-
SP0459	formate acetyltransferase (pfl)	1	6968302	0	0	0	0	1.00	0	0.00	0.00	-
SP0464	cell wall surface anchor family protein	0	0	0	0	0	0	-	0	-	-	-

SP0488	conserved hypothetical protein	4	24052159	0	0	457585	0	1.00	0	0.02	0.00	-
SP0547	conserved domain protein	3	21917644	0	0	0	0	1.00	0	0.00	0.00	-
SP0604	sensor histidine kinase VncS (vncS)	1	580342	371524	0	0	0	0.61	0	0.00	0.00	-
SP0694	conserved domain protein	4	0	0	0	0	0	-	0	-	-	-
SP0713	lysyl-tRNA synthetase (lysS)	4	240521667	0	0	0	0	1.00	0	0.00	0.00	-
SP0716	transcriptional regulator, putative	1	76790832	0	0	0	0	1.00	0	0.00	0.00	-
SP0721	conserved hypothetical protein	0	941741	659507	0	0	0	0.59	0	0.00	0.00	-
SP0723	conserved domain protein	3	63463180	0	0	0	0	1.00	0	0.00	0.00	-
SP0736	mannose-6-phosphate isomerase (manA)	0	234433333	2222475	0	0	0	0.99	0	0.00	0.00	-
SP0749	branched-chain amino acid ABC transporter; (livJ)	0	24610796	0	0	0	0	1.00	0	0.00	0.00	-
SP0789	conserved hypothetical protein	4	6676426	0	0	0	0	1.00	0	0.00	0.00	-
SP0805	hydrolase; haloacid dehalogenase-like family	4	0	0	0	0	0	-	0	-	-	-
SP0845	Lipoprotein	4	11214803	0	0	5027377	4344286	1.00	0	0.45	0.39	1.2
SP0851	conserved hypothetical protein	0	4505458	1487327	0	0	0	0.75	0	0.00	0.00	-
SP0860	pyrrolidone-carboxylate peptidase	4	46258840	0	0	0	0	1.00	0	0.00	0.00	-
SP0869	aminotransferase; class-V	3	86866992	2488646	0	0	0	0.97	0	0.00	0.00	-
SP0891	type I restriction-modification system, S subunit, putative	0	53117331	0	0	0	1247403	1.00	0	0.00	0.02	0.0
SP0937	conserved hypothetical protein	4	100607738	0	0	0	315729	1.00	0	0.00	0.00	0.0
SP0945	ribosome recycling factor (frr)	4	7194883	0	0	0	0	1.00	0	0.00	0.00	-
SP0954	competence protein CelA	2	32713657	700561	0	0	0	0.98	0	0.00	0.00	-
SP0962	lactoylglutathione lyase (gloA)	1	29199775	1003884	0	0	0	0.97	0	0.00	0.00	-
SP0964	dihydroorotate dehydrogenase B (pyrDB)	2	143362026	0	0	0	0	1.00	0	0.00	0.00	-
SP0979	oligoendopeptidase F (pepF)	2	0	1309304	0	0	0	-	0	-	-	-

SP0988	UDP-N-acetylglucosamine pyrophosphorylase	3	65766610	0	0	0	1879558	1.00	0	0.00	0.03	0.0
SP1102	conserved hypothetical protein TIGR00103	4	15637475	3714830	0	0	0	0.81	0	0.00	0.00	-
SP1118	pullulanase; putative	2	90590847	8499636	0	0	0	0.91	0	0.00	0.00	-
SP1132	hypothetical protein	4	38988761	0	0	0	0	1.00	0	0.00	0.00	-
SP1145	hypothetical protein	2	11185791	0	0	0	0	1.00	0	0.00	0.00	-
SP1249	guanosine monophosphate reductase (guaC)	4	258440000	3436079	0	1577798	0	0.99	0	0.01	0.00	-
SP1283	heat shock protein HtpX	0	2711680	0	0	0	0	1.00	0	0.00	0.00	-
SP1372	conserved hypothetical protein	4	15813701	0	0	0	1388225	1.00	0	0.00	0.09	0.0
SP1482	oxidoreductase; Gfo/Idh/MocA family	3	58810945	2746773	0	0	0	0.96	0	0.00	0.00	-
SP1504	TPR domain protein	2	665378	0	0	0	0	1.00	0	0.00	0.00	-
SP1524	aminotransferase; class II	3	23344438	0	0	0	0	1.00	0	0.00	0.00	-
SP1534	inorganic pyrophosphatase; manganese-dependent (ppaC)	2	189472205	751286	0	0	0	1.00	0	0.00	0.00	-
SP1542	asparaginyl-tRNA synthetase (asnS)	4	9085973	1607591	0	0	0	0.85	0	0.00	0.00	-
SP1555	dihydrodipicolinate reductase	4	201131667	2396188	0	0	0	0.99	0	0.00	0.00	-
SP1572	non-heme iron-containing ferritin	0	4532732	0	0	0	0	1.00	0	0.00	0.00	-
SP1631	threonyl-tRNA synthetase (thrS)	2	35983886	0	0	0	403799	1.00	0	0.00	0.01	0.0
SP1638	iron-dependent transcriptional regulator	4	52893321	810981	0	0	0	0.98	0	0.00	0.00	-
SP1650	manganese ABC transporter; (psaA)	4	9734070	0	17428897	27538234	1922867	1.00	1.8	2.83	0.20	14.3
SP1671	D-alanine--D-alanine ligase (ddlA)	4	71978671	0	0	0	0	1.00	0	0.00	0.00	-
SP1695	acetyl xylan esterase; putative	4	225571667	1387311	0	0	0	0.99	0	0.00	0.00	-
SP1699	holo-(acyl-carrier protein) synthase (acpS)	4	90436278	0	0	0	144595	1.00	0	0.00	0.00	0.0
SP1749	GTP-binding protein	2	15704080	337801	0	0	0	0.98	0	0.00	0.00	-
SP1752	mechanosensitive ion channel; putative	4	0	0	0	0	0	-	0	-	-	-

SP1782	ribosomal protein L11 methyltransferase (prmA)	4	215059232	0	0	0	0	1.00	0	0.00	0.00	-
SP1797	ABC transporter, permease protein	0	24326881	1154146	0	0	0	0.95	0	0.00	0.00	-
SP1802	hypothetical protein	4	0	0	0	0	0	-	0	-	-	-
SP1813	N-(5'-phosphoribosyl)-anthranilate isomerase (trpF)	4	0	0	0	0	0	-	0	-	-	-
SP1823	MgtC/SapB family protein	0	0	188071	0	0	0	-	0	-	-	-
SP1837	capsular polysaccharide biosynthesis protein; putative	1	26799826	0	0	0	0	1.00	0	0.00	0.00	-
SP1863	transcriptional regulator; MarR family	4	77881378	2771011	0	0	0	0.97	0	0.00	0.00	-
SP1872	iron-compound ABC transporter; iron-compound-binding protein	2	7011742	1091067	0	9834774	1535905	0.87	0	1.40	0.22	6.4
SP1910	conserved hypothetical protein	2	0	0	0	0	0	-	0	-	-	-
SP1918	ABC transporter; ATP-binding protein	1	2866801	0	0	0	0	1.00	0	0.00	0.00	-
SP1925	hypothetical protein	3	0	0	0	0	0	-	0	-	-	-
SP1959	nucleoside diphosphate kinase (ndk)	4	77516202	0	0	0	0	1.00	0	0.00	0.00	-
SP1978	diaminopimelate decarboxylase (lysA)	3	91265958	232244	0	0	0	1.00	0	0.00	0.00	-
SP1980	cmp-binding-factor 1	4	29898648	1855980	0	0	0	0.94	0	0.00	0.00	-
SP1983	ribulose-phosphate 3-epimerase	1	37216626	0	0	0	0	1.00	0	0.00	0.00	-
SP2039	conserved hypothetical protein	3	0	232085	0	0	0	-	0	-	-	-
SP2056	N-acetylglucosamine-6-phosphate deacetylase (nagA)	4	77564410	0	0	0	0	1.00	0	0.00	0.00	-
SP2105	hyphothetical protein	4	217858333	1437899	0	1084725	1379119	0.99	0	0.00	0.01	0.8
SP2129	PTS system; IIC component; putative	0	7561772	0	0	0	0	1.00	0	0.00	0.00	-
SP2132	conserved hypothetical protein	1	9556502	0	0	0	0	1.00	0	0.00	0.00	-
SP2174	D-alanyl carrier protein	4	9909577	0	0	0	0	1.00	0	0.00	0.00	-
SP2209	conserved hypothetical protein	1	0	0	0	0	0	-	0	-	-	-
SP2210	cysteine synthase (cysM)	3	19707901	0	0	0	0	1.00	0	0.00	0.00	-

The S-tag assay scores are correlated with the quantities of recombinant proteins in the soluble fraction after cell lysis, therefore the scores represent the solubility of the recombinant proteins (Kwon *et al*., 2007). The values of $H(F_{\text{His-tag}})$ represent fluorescence intensities from a labeled anti-His-tag antibody. Values of $E(F_{E.coli})$ and $S(F_{Sp})$ were obtained from fluorophore-labeled secondary antibodies, following binding of anti-*E.coli* and anti-*S.pneumoniae* antibodies, respectively. Values of $S_1(F_{27})$ and $S_0(F_{48})$ result from assays with a human patient antiserum and a healthy human antiserum, respectively.
